# Estimated global cancer incidence in the oldest adults in 2018 and projections to 2050

**DOI:** 10.1002/ijc.33232

**Published:** 2020-08-17

**Authors:** Sophie Pilleron, Enrique Soto‐Perez‐de‐Celis, Jerome Vignat, Jacques Ferlay, Isabelle Soerjomataram, Freddie Bray, Diana Sarfati

**Affiliations:** ^1^ Department of Public Health University of Otago Wellington New Zealand; ^2^ Department of Geriatrics, Cancer Care in the Elderly Clinic Instituto Nacional de Ciencas Médicas y Nutrición Salvador Zubirán Mexico City Mexico; ^3^ Cancer surveillance section International Agency for Research on Cancer Lyon France

**Keywords:** aged, cancer, epidemiology, incidence, older adults

## Abstract

Using GLOBOCAN estimates, we describe the estimated cancer incidence among adults aged 80 years or older at the regional and global level in 2018, reporting the number of new cancer cases, and the truncated age‐standardised incidence rates (per 100 000) for all cancer sites combined for this age group. We also presented the five most frequent cancers diagnosed by region and globally among females and males aged 65 to 79 years old and 80 years or older. We, finally, estimated the number of new cancer cases in 2050, the proportion of cases aged 80 years or older, and the proportional increase between 2018 and 2050 by region, by applying population projections to the 2018 incidence rates. In 2018, an estimated 2.3 million new cancer cases (excluding nonmelanoma skin cancers) were aged 80 years or older worldwide (13% of all cancer cases), with large variation in the profiles at regional levels. Globally, breast, lung and colon were the most common cancer sites diagnosed in the oldest females, while prostate, lung and colon were most frequent in the oldest males. In 2050, an estimated 6.9 million new cancers will be diagnosed in adults aged 80 years or older worldwide (20.5% of all cancer cases). Due to the complexity of cancer management in the oldest patients, the expected increase will challenge healthcare systems worldwide, posing a tangible economic and social impact on families and society. It is time to consider the oldest population in cancer control policies.

## Abstract 2

**What's new?**

The global population of aging persons is expected to increase significantly over the coming decades. To prepare for the forthcoming challenges in cancer care, more information is needed on cancer burden among the world's oldest patients. Here, assessment of global cancer burden in adults in 2018 shows that 2.3 million new cases occurred in persons aged 80 or older that year, accounting for 13 percent of all cases worldwide. Projections suggest that by 2050, 7 million new cancer cases will affect this age group. The findings predict substantial challenges for healthcare and highlight the need for aging‐specific cancer control initiatives.

**Abbreviations**
IARC: International Agency for Research on Cancer
TASR: Truncated age‐standardised incidence rate

## 1 INTRODUCTION

The number of people aged 80 years or older is expected to triple by 2050 worldwide, from 143 million in 2019 to 426 million by 2050,^1^ an increase largely driven by population ageing and growth.^2^ However, little is known about the global variations in the magnitude and profiles of cancer among the oldest old, with the few studies that exist mainly from Europe and the U.S.^3^, ^4^

Cancer management in the oldest patients can be complex, given the high level of comorbidity, frailty, decline of functional status and limited life expectancy affecting this age group.^5^ As the common exclusion of patients with cancer aged over the age of 65 from clinical trials^6^ and the considerable heterogeneity in terms of health status and fitness among the oldest population, both undertreatment and overtreatment remain a concern.^7^ As a consequence, the oldest patients have the lowest cancer‐specific survival relative to other age groups,^8^, ^9^, ^10^ and the survival gap is widening, partially because the oldest patients do not benefit as much from advances in cancer treatment as younger patients.^8^, ^9^

In view of an unprecedented rising number of future patients in this age group and the challenges of their cancer management, a comprehensive description of the cancer burden in the oldest population is warranted. We thus provide a detailed profile of the current and future cancer estimated burden in the oldest adults worldwide and by world region, as a call for the design of dedicated and tailored cancer control programs for populations aged 80 years and above.

## 2 METHODS

While the definition of an oldest‐old person may vary according to the life expectancy of a given country, the *United Nations’ World Population Ageing* report defines the oldest population as those aged 80 years and over.^11^

Population‐based cancer incidence estimates for the year 2018 were obtained for major cancer sites and all cancer sites combined (excluding nonmelanoma skin cancers) for 185 countries and world regions, by sex and age group (80‐84, 85 and over) from the GLOBOCAN database held at the International Agency for Research on Cancer (IARC).^2^ The method used to obtain GLOBOCAN estimates was fully described elsewhere.^2^ In brief, the GLOBOCAN national estimates are based ideally on the availability of recorded high‐quality national and subnational incidence (from population‐based cancer registries) and national mortality data (from vital registration systems). In countries where national mortality data were available but national or subnational cancer registries were not, national incidence estimation relied on national mortality estimates and modelling of the mortality to incidence ratio from neighbouring countries. In countries where neither mortality nor incidence data are available, incidence estimates was based on neighbouring countries.

We reported the numbers of new cancer cases among adults aged 80 years or older and the truncated age‐standardised incidence rates (TASR per 100 000) for all cancer sites combined for the 185 countries and the following six United Nations geographic regions and their subregions (in parentheses): Africa (Eastern Africa, Middle Africa, Northern Africa, Southern Africa and Western Africa), Asia (China, Eastern Asia excluding China, South Central Asia, South‐Eastern Asia and Western Asia), Europe (Central Eastern Europe, Northern Europe, Southern Europe and Western Europe), Latin America and the Caribbean (Caribbean, Central America and South America), Northern America and Oceania (Australia/New Zealand, Melanesia and Micronesia/Polynesia; https://population.un.org/wpp/DefinitionOfRegions/). The TASRs in adults aged 80 years or older were calculated from the rates of 80 to 84 and 85+ age groups, using the standard world population of Segi revised by Doll et al.^12^ We also computed the number of new cancer cases occurring among adults aged 80 years or older as a proportion of the total number of cancer diagnoses (all ages combined). We presented the number of new cases of the five most common cancer sites as the percentage of total new cancer cases diagnosed among adults aged 65 to 79 years old and those aged 80 years or older by sex for all regions. We showed China and India separately because of their large population size. Finally, we predicted the number of new cancer cases (all cancer sites) among adults aged 80 years or older that will occur in 2050 for all world regions by applying the age‐specific rates in 2018 to the corresponding national population projections in 2050 obtained from the United Nations Population Division.^1^ In our projections, we considered only the effect of ageing and population growth and assumed no change in the risk pattern of cancer incidence between 2018 and 2050.^13^, ^14^

## 3 RESULTS

### 3.1 Global cancer incidence in the oldest adults

In 2018, 2.3 million estimated new cancer cases occurred in adults aged 80 years or older worldwide, representing 13.3% of all global new cancer cases diagnosed in 2018. This proportion significantly varied across regions, from 6% in Africa to 18% in Europe and Oceania (Table 1). At the country level, Japan had the highest percentage of total cancer cases occurring among adults aged 80 years or older (31%), while the Solomon Islands had the lowest percentage in the world (2%; Table S1).

**TABLE 1:** Estimated number of new cancer cases in adults aged 80 or older, percentage of total cases (all age combined), percentage of the total population aged 80 years or older and truncated age‐standardised incidence rates (TASRs), 2018

Regions and subregions	% of population aged 80+	Estimated number of cases among 80+	% of total cases	TASRs (per 100 000)
Africa	0.5	60 200	5.8	967
Eastern Africa	0.4	16 500	5.1	861
Middle Africa	0.4	5500	5.8	850
Northern Africa	0.9	20 700	7.4	950
Southern Africa	0.8	9600	8.8	1883
Western Africa	0.3	7800	3.5	792
Asia	1.5	984 500	11.4	1429
Eastern Asia excludes China	5.9	318 100	24.0	2351
China	1.9	437 000	10.2	1622
South Central Asia excludes India	0.9	40 500	7.1	745
India	1.0	72 300	6.3	550
South‐Eastern Asia	1.1	77 400	7.9	1038
Western Asia	1.0	39 100	10.0	1402
Europe	5.2	711 100	18.2	1844
Central‐Eastern Europe	4.0	146 000	12.1	1229
Northern Europe	5.0	133 600	21.4	2541
Southern Europe	6.4	180 400	20.7	1853
Western Europe	6.1	251 100	20.7	2116
Latin America and the Caribbean	1.8	183 000	13.6	1559
Caribbean	2.4	16 600	15.6	1562
Central America	1.5	30 800	12.5	1109
South America	1.9	135 600	13.7	1717
Northern America	3.9	301 600	15.9	2133
Oceania	3.1	32 300	17.9	2557
Australia/New Zealand	4.0	31 200	19.0	2633
Melanesia	0.6	900	6.2	1400
Micronesia/Polynesia	1.2	200	9.0	1559
World	1.9	2 272 700	13.3	1613

With an estimated 984 500 new cancer cases (43% of the global figure), Asia was the region with the highest number of new cancer cases in adults aged 80 years or older. Europe ranked second with an estimated 711 100 new cancer cases in this population or 31% of the global burden. With an estimated 437 000 new cancer cases, China alone comprised 44% of cases among adults aged 80 years and older in Asia, and 19% of the new cases globally.

**FIGURE 1:**
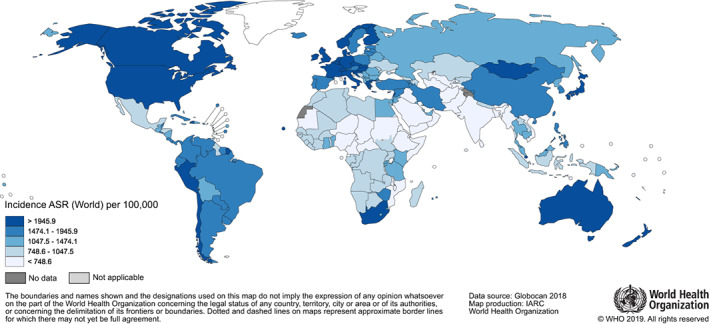
Truncated age‐standardised incidence rates (TASR) in adults aged 80 years or older for both sexes combined in the world in 2018 [Color figure can be viewed at wileyonlinelibrary.com]

Truncated age‐standardised incidence rates also varied greatly across regions, from 967 per 100 000 adults aged 80 years or older in Africa to 2557 in Oceania, and within regions (Table 1 and Figure 1). As examples, regional TASRs ranged from 607 in South‐Central Asia to 2351 in Eastern Asia (excluding China), and nationally from 275 in The Gambia to 3615 in Singapore (Table S1).

### 3.2 Cancer profile in the oldest adults

**FIGURE 2:**
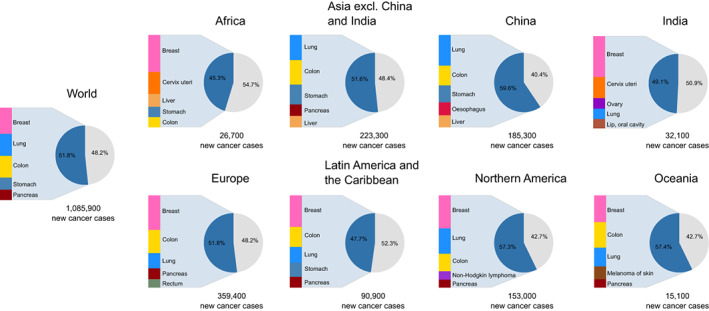
The five most common cancer diagnosed among females aged 80 years or older by world region plus China and India in 2018 [Color figure can be viewed at wileyonlinelibrary.com]

Among females aged 80 years or older, breast, lung and colon cancers were the most common cancer sites globally and in most regions (Figure 2). Stomach cancer was frequent in Africa, Asia and Latin America and the Caribbean; cervical and liver cancers in Africa. In Asia, lung cancer ranked first, and breast cancer was not a leading cancer, specifically in China where it ranked eighth. In India, the ovary cancer and the cancers of the lip and the oral cavity were part of the five most common cancers. The cancer profile in females aged 80 or older globally is similar to that of females aged 65 to 79 years except for Asia, where breast cancer ranked first and second in the 65 to 79 age group for Asia excluding India and China, respectively (Figure S1). The five most common cancers represented 52% of the total number of new cancer cases that occurred in the oldest females in 2018 worldwide, ranging from 45% in Africa to 60% in China.

**FIGURE 3:**
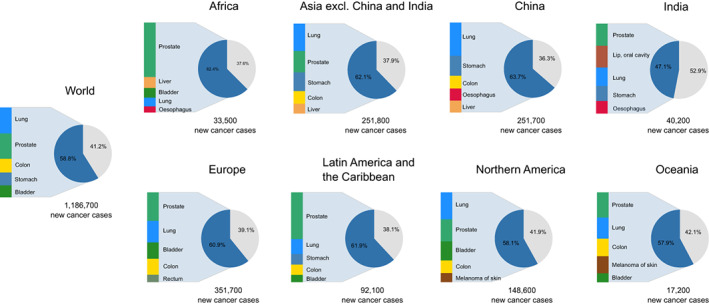
The five most common cancer diagnosed among males aged 80 years or older by world region plus China and India in 2018 [Color figure can be viewed at wileyonlinelibrary.com]

Among males aged 80 years or older, lung and prostate cancers were the leading cancer sites at the global level (Figure 3). Colon cancer was frequent in almost all regions. Stomach cancer was frequent in Asia and Latin America and the Caribbean and ranked fourth at the global level. Contrary to other regions, liver cancer was common in Africa (second) and Asia (fifth). The cancer profile in the 80 years or older age group is also similar to that observed in the 65 to 79 age group (Figure S2). Globally, the five most common cancers represented 59% of all cancers diagnosed in the oldest males, varying from 58% in Northern America and Oceania to 64% in China.

### 3.3 Projections of cancer incidence by 2050 in the oldest adults

By 2050, an estimated 6.9 million new cancer cases (21.5% of global cases, all ages combined) are expected to be diagnosed in adults aged 80 years or older worldwide. Over a quarter of the global new cancer cases (27%) will occur in China alone, with a further 26% in Asia and 19% in Europe.

**FIGURE 4:**
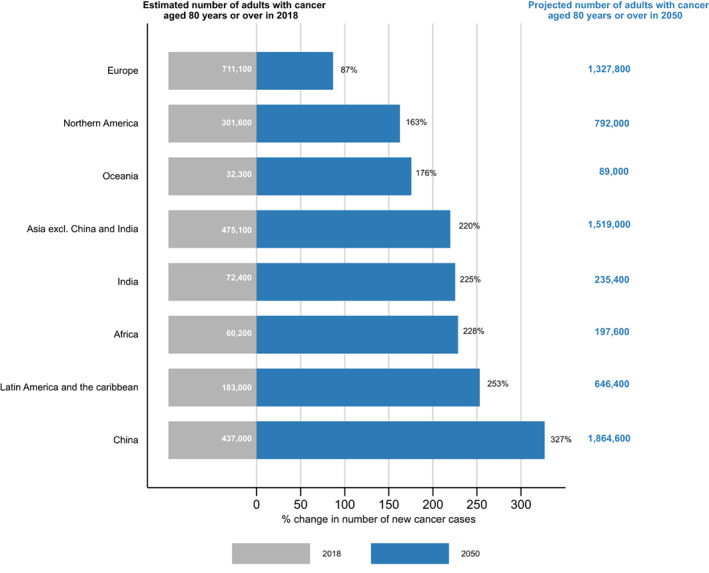
Percentage change in the number of new cancer cases among adults aged 80 years or older by 2050 by world region plus China [Color figure can be viewed at wileyonlinelibrary.com]

An increase of over 200% compared to the 2018 figure is expected at the global level. The most significant increases are expected in China (+327%), followed by Latin America and the Caribbean (+253%), and Africa (+228%), while the lowest increase will be seen in Europe (+87%, Figure 4).

## 4 DISCUSSION

To the best of our knowledge, no attempt to describe the cancer epidemiology among adults aged 80 years or older has been made at the global level. Over 2 million estimated cancers were diagnosed in adults aged 80 years or older in 2018, with breast, lung, colon and stomach cancers the most common types among females, and prostate, lung and colon cancer, the leading types among males. Due to population ageing, the estimated number of new cancer diagnoses is expected to triple by 2050 worldwide. Given the complexity of cancer management in the most aged patients, this increase will challenge already strained healthcare systems worldwide. Our study advocates for a better consideration of the oldest population by clinicians, policymakers and researchers to reduce the substantial burden that cancer will progressively affect the oldest adults and their families over the next decades.

The high level of comorbidity, frailty, age‐related physiological changes and average life expectancy in the oldest patients may complicate their cancer management. Also, patients aged 80 years and over are seldom included in randomised clinical trials, resulting in a lack of knowledge about the benefit/risk ratio of treatment strategies.^15^ Because the oldest adults are heterogeneous in terms of health status, and fitness, chronologic age alone is often a poor indicator of an individual's physiological or functional status, and should not be used as the sole criterion for treatment decision‐making.^16^, ^17^ Cancer survival is lower in this age group compared to other age groups^8^, ^9^, ^10^, ^18^; which is mainly explained by excess mortality in the first months after diagnosis,^9^, ^18^ possibly due to suboptimal treatment, higher postoperative mortality, inappropriate risk stratification.^16^, ^19^, ^20^ Because some older patients may benefit from surgery or chemotherapy,^21^, ^22^, ^23^ individualised cancer management (including, but not limited to, modification of treatment schedules and/or dosing and implementation of geriatric‐specific supportive measures) is, therefore, essential in the oldest adults. The utilisation of comprehensive geriatric assessments is crucial to identifying patients that will optimally benefit from treatment, and in countering other deficits that could lead to improved treatment tolerance.^16^, ^24^

Less developed countries face specific challenges, given a substantial increase in the number of new cancer diagnoses among their oldest populations. In addition to a lack of specialised infrastructure, oncologists, pathologists and surgeons, and lack of availability of, or accessibility to radiotherapy and chemotherapy,^25^, ^26^ there is also a generalised lack of cancer care personnel with geriatric expertise, and geriatric oncology is vastly underdeveloped.^27^ Because of competing health demands, the oldest adults may not currently represent a priority in cancer plans and programs in resource‐limited countries. However, due to the predicted rise in the number of patients diagnosed with cancer, it is clear that these countries should consider the specific needs of the oldest adults when developing and implementing cancer control programs.

Supportive and palliative care, including pain management, are crucial to relieve unnecessary pain and suffering for patients and their families, and are, therefore, essential components of cancer care management. Investment in palliative care is cost‐effective for the healthcare system and society regardless of the level of country development.^28^ Currently, supportive/palliative care and pain medications are not available in many of the least developed countries,^29^ and even where they are, older age is a barrier to their implementation.^30^ Although pain medications, such as opioids, can be delivered to the majority of patients regardless of chronological age, the oldest patients with cancer warrant careful clinical consideration when managing their pain because of age‐related physiologic changes, immunosuppression, polypharmacy, comorbidity and frailty.^30^ Though international organisations have issued guidelines for palliative care and pain management,^31^ specific considerations regarding older adults still need to be addressed, and this represents a relevant gap for future research.

Alongside the challenges for healthcare systems, the projected rise in the number of patients with cancer aged 80 years or older will have a major social and economic impact on families and society. The oldest adults with cancer often experience a decline in functional status^32^, ^33^; therefore, they need support, usually from within the family, to take care of them to undertake daily activities. The caregiver role is recognised as central in cancer care management.^34^ Yet caregiver burden defined as “*the strain or load borne by a person who cares for a chronically ill, disabled, or elderly family member*”,^35^ is common in persons who care for older patients,^36^, ^37^ and is associated with higher psychosocial and physical morbidity.^38^, ^39^ Although a growing number of effective psychosocial interventions to reduce caregiver burden and its negative effects have been developed in high‐resource countries,^34^ little is known about the caregiver burden among persons who care for a relative with cancer in resource‐limited settings. Along with appropriate consideration of the oldest patients with cancer in cancer control, policies should also acknowledge and address caregiver burden as an important global health care issue.

A sizable proportion of cancers among the oldest adults are preventable. From a life‐course perspective, the current cancer profile observed among the oldest population results from exposure to risk factors in the previous decades. It is then likely that decreasing exposure to risk factors (ie, smoking, alcohol consumption, obesity, human papillomavirus, hepatitis C) over the entire lifespan will have an impact on the cancer burden and also on comorbidity in the future oldest cohorts. Some studies have shown that primary prevention, such as smoking cessation, dietary modification or exercise, remains possible after the age of 65 years.^40^, ^41^, ^42^, ^43^ Even though there is a paucity of evidence in the oldest adults, one can assume that encouraging smoking cessation, reducing alcohol intake and increasing physical activity may also lower the risk of cancer among the oldest people. Acting to prevent cancer over the entire lifespan is the only sustainable and cost‐effective way to reduce cancer burden at all ages, including the oldest old, particularly in resource‐limited countries.

In terms of secondary prevention, organised cancer screening programs are not designed to target adults aged 80 years or older because of a lack of evidence of their effectiveness at the oldest age and the limited life expectancy gains among this subpopulation.^44^ However, some screening guidelines state that, in selected cases, individual screening may be pursued over the age of 75 after thoughtful consideration of life expectancy, patient's preferences and the balance of benefit/harm by the practitioner.^44^, ^45^ Nevertheless, older patients may still undergo screening despite a limited life expectancy, leading to overdiagnosis of indolent disease. In the US, for example, up to 40% of men aged 80 years or older were screened with prostate‐specific antigen (PSA) in 2013, despite recommendations to the contrary.^46^ However, the proportion of prostate cancer cases among men aged 80 years or older which are a result of overdiagnosis has been estimated to be of only 6% (compared to 32% in those aged 70‐74 years).^47^ In addition, recent data suggests that global prostate cancer incidence has decreased, which might reflect declines PSA screening.^48^ Therefore, we believe that the effect of overdiagnosis on our results may not be as significant, and this may apply not only to prostate cancer, but also for other screen‐detected tumours such as colon and breast cancer. Irrespectively, increasing access to breast, colorectal and cervical cancers screening for the current targeted age group (ie, up to 74‐years‐old for colorectal cancer in some countries) might also benefit the oldest cohorts in a long‐term perspective.

Our study has limitations. In countries with no incidence data, GLOBOCAN estimates were computed from cancer specific mortality data that may be less accurate for older age groups; where vital statistics system exists, mortality data rely on the accuracy of the cause of death reported on the death certificate. Identifying the underlying cause of death may be challenging in older patients who may present with fatal comorbidities. In many low resource countries, verbal autopsy is the only method currently available to obtain estimates of the distribution of causes of death but the method may be less reliable in older age groups.^49^Furthermore, there is a higher probability of under‐ascertainment of cancer cases at older ages because of comorbidities and frailty, as well as limited histological verification of cancer diagnoses among the oldest‐old patients.^4^ The actual incidence may then be higher than our estimation. Besides, our projections neither took into account historical trends in cancer incidence or potential changes in risk in younger cohorts, nor preventive actions taken to tackle the cancer burden in each region. Cancer‐specific projections for 2050 and the impact of various cancer prevention interventions on 2050 projections were beyond the scope of this report. However, our estimates for all cancer sites combined at global and regional levels in 2050 are probably underestimated. Indeed, countries in transition are seeing the incidence of cancers common in the oldest population, naming breast, colorectal and prostate cancers, rising. In the same time, the decrease of infection‐related cancers, notably in gastric and cervical cancers, are decreasing in high‐resourced countries.^50^Finally, the definition of oldest‐old might vary according to the life expectancy of populations, and it is possible that setting the cut‐off at 80 years might be too high for less developed regions of the world.

## 5 CONCLUSION

The substantial rise in cancer cases among the oldest adults in the next decades represents a considerable challenge for healthcare systems across all world regions. Our study highlights the need for prioritising cancer prevention over the entire lifespan and the inclusion of the oldest adults in cancer control programs through the creation of age‐friendly healthcare systems and of multidisciplinary teams with geriatric expertise and training which can provide high‐quality care for this growing population of patients with cancer.

## CONFLICT OF INTEREST

The authors declare that they have no conflicts of interest.

6

## Supporting information


**Appendix S1**: Supporting information

## Data Availability

The data that support the findings of our study are openly available within the Global Cancer Observatory at http://gco.iarc.fr for the 2018 GLOBOCAN estimates.^2^ Datasets used for the analysis will be made available upon reasonable request.
