# Supplementary material for: Estimated global cancer incidence in the oldest adults in 2018 and projections to 2050
Source: Int J Cancer. 2020 Aug 17;148(3):601–8. doi: 10.1002/ijc.33232 (PMC7754149; doi:10.1002/ijc.33232)
Supplement: Supplementary file 1 — Appendix S1: Supporting information [file IJC-148-601-s001.pdf]

Global cancer incidence in the oldest adults in 2018 and projections to 2050

Sophie Pilleron, Enrique Soto-Perez-de-Celis, Jerome Vignat, Jacques Ferlay, Isabelle Soerjomataram, Freddie Bray, Diana Sarfati

Table of content

Supplementary Tables

Supplementary Table. Number of new cancer cases in adults aged 80 or older, percentage of total cases (all age combined), percentage of the total population aged 80 years or older, and age-standardised incidence rates (ASRs) by country, 2018

Supplementary Figures

Supplementary Figure 1. Cancer profile in females aged 65-79 years by world region, 2018

Supplementary Figure 2. Cancer profile in males aged 65-79 years by world region, 2018

Supplementary Table. Number of new cancer cases in adults aged 80 or older, percentage of total cases (all age combined), percentage of the total population aged 80 years or older, and age-standardised incidence rates (ASRs) by country, 2018

Country	New cancers cases among 80+	% total new cancer cases	% total population aged 80+	ASRs
Afghanistan	500	2.8	0.3	548
Albania	1,000	13.0	2.8	1,250
Algeria	4,200	8.2	1.3	797
Angola	800	5.4	0.3	865
Argentina	18,800	15.1	2.8	1,491
Armenia	1,600	18.8	3.3	1,654
Australia	26,500	19.1	4.1	2,634
Austria	8,000	19.0	5.4	1,715
Azerbaijan	1,000	9.3	1.4	773
Bahamas	100	9.0	1.8	1,117
Bahrain	100	5.6	0.3	1,148
Bangladesh	10,700	7.2	1.1	592
Barbados	100	15.2	3.6	1,845
Belarus	4,500	11.1	4.0	1,175
Belgium	14,000	19.5	5.7	2,142
Belize	40	11.4	0.6	1,681
Benin	500	6.9	0.4	1,112
Bhutan	100	8.8	1.0	622
Bolivia	2,000	14.2	1.6	1,162
Bosnia Herzegovina	1,500	11.4	4.4	958
Botswana	100	6.7	0.5	1,032
Brazil	65,200	12.4	1.8	1,714
Brunei Darussalam	100	8.7	0.7	2,439
Bulgaria	4,000	12.1	4.8	1,203
Burkina Faso	300	3.5	0.2	832
Burundi	400	5.1	0.4	1,059
Cambodia	1,000	7.0	0.6	1,146
Cameroon	700	5.1	0.5	704
Canada	35,800	17.7	4.4	2,214
Cape Verde	100	27.8	1.3	2,812
Central African Republic	100	6.5	0.5	671
Chad	200	3.8	0.3	578
Chile	9,600	18.9	2.6	2,036
Colombia	12,500	12.9	1.5	1,724
Comoros	0	3.7	0.4	512
Congo, Democratic People Republic of	3,000	6.3	0.4	971
Congo, Republic of	200	8.9	0.5	759
Costa Rica	1,900	16.8	2.1	1,888
Cote d Ivoire	700	4.9	0.3	1,003
Croatia	4,400	18.6	5.5	1,952
Cuba	7,100	16.9	3.9	1,573
Cyprus	700	16.7	3.1	2,088
Czechia	8,800	14.4	4.1	2,024
Denmark	6,200	16.5	4.5	2,408
Djibouti	0	2.9	0.6	309
Dominican Republic	2,400	13.5	1.7	1,285
Ecuador	4,900	18.3	1.6	1,890
Egypt	9,400	7.4	0.8	1,213
El Salvador	2,200	22.4	2.0	1,874
Equatorial Guinea	0	3.9	0.4	564

Eritrea	100	4.6	0.5	529
Estonia	1,400	18.9	5.7	1,891
Ethiopia	2,200	3.4	0.5	409
Fiji	0	3.2	0.7	718
Finland	6,600	21.2	5.4	2,229
France (metropolitan)	83,600	20.3	6.2	2,064
France, Guadeloupe	300	16.7	4.8	1,678
France, La Reunion	400	14.3	2.6	1,741
France, Martinique	300	18.8	5.5	1,723
France, New Caledonia	100	13.9	2.0	2,917
French Guyana	0	8.2	0.8	2,041
French Polynesia	100	8.1	1.3	1,727
Gabon	100	9.2	0.9	842
Georgia	1,200	13.4	3.8	823
Germany	118,500	22.3	6.6	2,196
Ghana	1,400	6.4	0.4	1,119
Greece	14,700	22.8	6.7	1,993
Guam	100	14.7	2.0	1,779
Guatemala	2,400	15.3	1.1	1,373
Guinea	300	5.5	0.3	980
Guinea-Bissau	40	4.1	0.3	788
Guyana	100	8.8	0.8	1,019
Haiti	1,900	16.1	0.8	2,280
Honduras	900	9.4	1.0	937
Hungary	8,900	13.4	4.3	2,118
Iceland	200	16.6	3.8	1,853
Indonesia	15,300	4.5	0.7	784
Iran, Islamic Republic of	13,000	12.2	1.0	1,632
Iraq	1,100	4.7	0.5	625
Ireland	3,800	14.9	3.2	2,535
Israel	4,700	17.7	3.1	1,820
Italy	88,600	23.4	7.4	2,034
Jamaica	1,000	15.0	2.6	1,474
Japan	266,800	30.5	8.4	2,504
Jordan	800	7.7	0.6	1,417
Kazakhstan	2,200	6.8	1.4	808
Kenya	2,500	5.5	0.4	1,182
Korea, Democratic People Republic of	4,400	8.0	1.6	1,081
Korea, Republic of	30,600	11.2	3.2	1,840
Kuwait	100	3.5	0.2	1,403
Kyrgyzstan	400	7.7	1.0	787
Lao People Democratic Republic	400	5.3	0.6	981
Latvia	2,000	17.4	5.6	1,916
Lebanon	2,600	15.6	1.8	2,416
Lesotho	200	11.7	0.7	1,393
Liberia	100	5.0	0.3	899
Libya	400	7.8	0.8	978
Lithuania	2,700	17.3	5.7	1,682
Luxembourg	500	19.2	4.1	2,358
Macedonia	600	8.4	2.5	1,200
Madagascar	900	5.3	0.4	846
Malawi	300	2.0	0.4	519
Malaysia	3,400	7.9	1.0	1,096
Maldives	20	5.7	0.9	585
Mali	400	3.4	0.3	853
Malta	300	17.7	4.1	2,242
Mauritania	100	4.6	0.4	741

Mauritius	300	12.2	2.0	1,360
Mexico	20,700	11.4	1.6	985
Moldova	900	6.3	2.3	956
Mongolia	400	7.3	0.6	2,075
Montenegro	200	11.3	3.5	1,124
Morocco	3,300	6.5	1.2	791
Mozambique	800	3.6	0.4	670
Myanmar	2,900	4.4	0.8	675
Namibia	100	5.6	0.5	912
Nepal	2,000	8.1	0.8	935
New Zealand	4,600	18.5	3.7	2,624
Nicaragua	1,000	13.1	1.3	1,237
Niger	200	2.9	0.2	476
Nigeria	2,300	2.0	0.2	534
Norway	5,600	17.7	4.2	2,537
Oman	100	3.3	0.4	553
Pakistan	6,500	3.9	0.7	474
Palestine	400	10.4	0.4	2,319
Panama	1,400	18.4	1.9	1,773
Papua New Guinea	600	6.0	0.6	1,399
Paraguay	1,300	12.4	1.3	1,569
Peru	10,900	17.1	1.6	2,179
Philippines	12,500	8.9	0.7	1,763
Poland	28,000	15.4	4.4	1,672
Portugal	11,400	20.6	6.4	1,739
Puerto Rico	2,200	15.9	4.0	1,552
Qatar	50	4.3	0.2	1,134
Romania	11,600	14.2	4.7	1,240
Russian Federation	60,000	11.4	3.8	1,076
Rwanda	600	6.0	0.4	1,216
Saint Lucia	50	13.0	2.5	1,055
Samoa	30	8.5	1.2	1,191
Sao Tome and Principe	10	9.3	0.7	642
Saudi Arabia	1,200	4.9	0.5	735
Senegal	500	5.5	0.4	953
Serbia	3,700	8.0	3.9	1,051
Sierra Leone	100	2.8	0.2	830
Singapore	5,600	21.8	2.7	3,615
Slovakia	3,700	13.1	3.2	2,099
Slovenia	2,300	17.7	5.4	2,062
Solomon Islands	10	1.8	0.5	283
Somalia	200	3.0	0.3	609
South Africa	9,000	8.9	0.8	1,954
South Sudan	400	5.0	0.4	812
Spain	50,100	20.1	6.3	1,720
Sri Lanka	1,800	7.8	1.6	536
Sudan	1,400	5.5	0.5	694
Suriname	100	12.3	1.3	1,636
Swaziland	80	7.7	0.4	1,452
Sweden	9,900	17.8	5.2	1,936
Switzerland	8,600	19.1	5.2	1,960
Syrian Arab Republic	2,100	9.3	0.8	1,476
Tajikistan	400	7.9	0.8	631
Tanzania	3,000	7.4	0.5	1,110
Thailand	21,800	13.0	2.5	1,267
The Gambia	10	2.0	0.3	275
The Netherlands	17,400	16.7	4.7	2,177
Timor-Leste	30	4.4	0.4	560

Togo	100	3.9	0.3	826
Trinidad and Tobago	300	10.0	1.8	1,375
Tunisia	1,700	11.6	1.7	914
Turkey	20,900	10.3	1.6	1,583
Turkmenistan	300	5.4	0.9	594
Uganda	1,400	4.4	0.3	1,032
Ukraine	15,200	9.3	4.2	782
United Arab Emirates	100	3.2	0.1	1,249
United Kingdom	94,300	23.5	5.2	2,745
United States of America	265,600	15.7	3.8	2,122
Uruguay	2,900	20.6	4.4	1,910
Uzbekistan	2,100	8.6	1.0	666
Vanuatu	10	4.5	0.5	645
Venezuela	6,800	11.7	1.3	1,623
Viet Nam	14,000	8.6	2.1	708
Yemen	600	4.6	0.3	609
Zambia	600	5.5	0.4	961
Zimbabwe	1,600	9.4	0.5	1,894

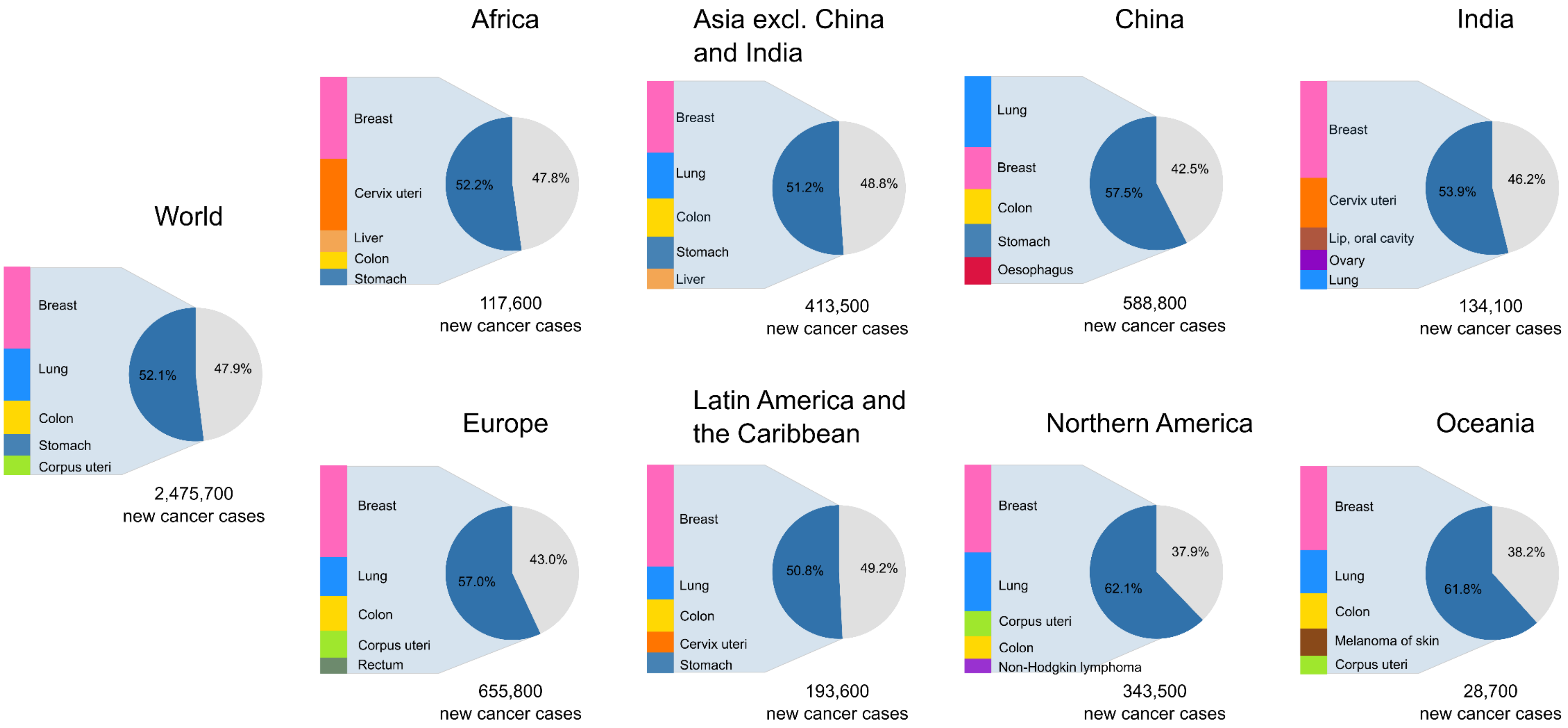

Supplementary Figure 1. Cancer profile in females aged 65-79 years by world region, 2018

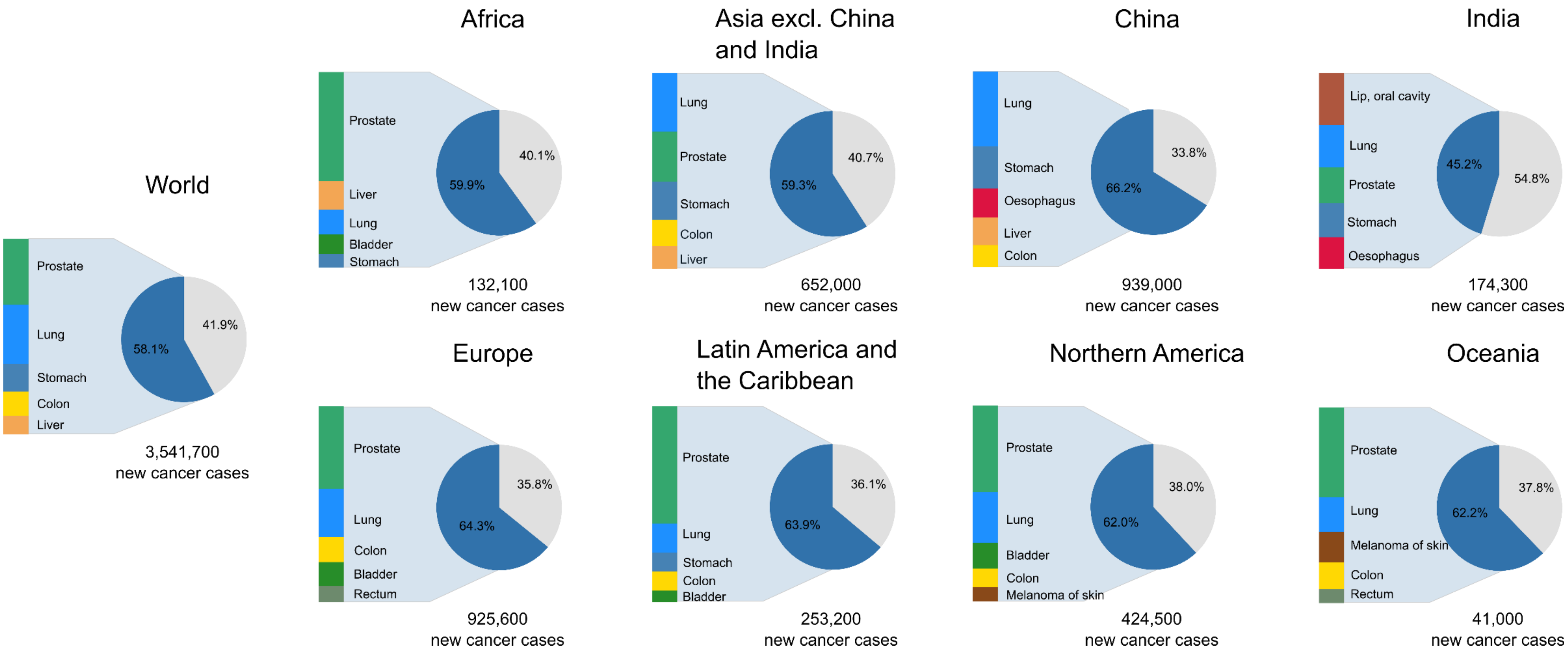

Supplementary Figure 2. Cancer profile in males aged 65-79 years by world region, 2018
